# Lunacy in Scotland

**Published:** 1902-08-30

**Authors:** 


### LUNACY IN SCOTLAND.

The 44th annual report of the Commissioners in Lunacy for Scotland, recently issued, shows that on January 1st, 1902, there were 16,288 insane persons in Scotland of whom the Commissioners had official cognisance, but these figures included the inmates of the training schools for imbecile children, and also the criminal lunatics in the prison at Perth. Of the above-named total 2,401 were maintained as private patients, 13,841 were on the rates, and 46 were paid for directly by the State. The total shows that the insane population of Scotland had increased by 389 during the year.

The Commissioners in Lunacy entered on their duties in 1858, and since that date the private patients have increased by 1,232, the training schools by 368, and the criminal lunatics by 20. At first sight the increase of all classes of the insane seems very alarming, and not much consolation can be obtained by looking more closely into the figures, for we find it stated that while the number of lunatics under jurisdiction of the board has, since 1858, increased by 180 per cent., the population of the country has only increased by 49 per cent. And it is further pointed out that the increase during 1901 has been 84 in excess of the average for the previous ten years. In the year 1892 the proportion of pauper lunatics to every 100,000 of the population in Scotland was only 203, whereas it is now 244 to every 100,000. Although qualifying circumstances might be mentioned, the general reader may well ask when the monotonous tale of the annual increase in the numbers of the insane is to stop. The increment, too, is an all-round one, for we find it in the royal asylums, in the district asylums, in parochial asylums and in private institutions, and in poor-houses.

Voluntary patients are received into the Scottish asylums on a simple application signed by the patient; but such patients cannot be detained more than three days after giving notice of their intention to leave. During 1901 the number of voluntary patients was 90, and at the end of the year the number under treatment was 75. Such patients are seen by the Commissioners at their annual visits, and the Commissioners say that no difficulty has been traced to the presence of these patients in asylums; and that they are of opinion that their reception is a useful provision of the law. In the year 1901 the average death-rate in the Scottish asylums was 8·4 per cent. of the average number resident, being ·6 per cent. lower than it was in 1900.

The recoveries in royal and district asylums were 39 per cent. of the admissions, in private asylums 43, and in parochial asylums 51 per cent. Taken together, these percentages are lower than in 1900. We find that 152 patients were liberated on probation, and the Commissioners remark that sometimes patients who appear well enough for discharge when in the asylum become unsettled when the influence of the asylum is removed; but the large majority so liberated undergo no deterioration, and many are benefited.

A page of the report is occupied by the eternal question of changes among the staff of attendants and nurses, and it is shown that the most of these changes occur among those who have only been a short time in the asylum. It might be inferred from this that the inducements to enter the service are sufficient, but that the conditions inseparable from the service are not always conducive to a lengthened stay. Verily an attendant or nurse has many unpleasant duties to perform from time to time which may repel some of them; but many of the conditions are pleasant enough, and we believe the Commissioners have hit the right nail on the head when they refer to the pensions which ought to be forthcoming to every asylum attendant and nurse after long and faithful service. Careless people at the age of 25 or so think little of pensions not obtainable for a quarter of a century; but the careful man or woman thinks much of this assured provision for old age, and it is the careful element that should be encourged by certainty of pensions. We are glad that the Scottish Commissioners have taken the matter up, and we hope they will not stop until the question is finally and satisfactorily settled.

One remarkable fact which must always strike the reader of these Blue-Books is the relatively large proportion of lunatic paupers who are boarded out, either with their own relatives or with strangers; and to us it is a constant source of wonder that this system, which has borne the test of experience in Scotland, should be so little used in England to reduce the enormous number of our asylum lunatics. The accounts of the visitation of these patients by the Deputy Commissioners are almost always satisfactory. One village contains 58 patients, and seven other villages have an aggregate of 122 patients. The Commissioners say that these patients generally would compare favourably as regards robust appearance with any in Scotland. "They share everything equally with the family." At Benderloch in Argyllshire there is a small colony of male patients, and we are told that these patients were "all in good physical condition, usefully employed, and none of them had any complaint to make as to their diet and treatment."

Just as in England, so is there in Scotland a deficiency of asylum accommodation for private patients of the lower middle class. The Commissioners advise that adjuncts for these cases should be built at the district asylums, and they state their belief that the rates would thereby be relieved. There cannot be a doubt that this is a correct view. The cost of the pauper lunatics ranges from £24 to £33 per annum in the royal asylums, and from £18 5s. to £34 in the district asylums.

In our notices of these reports in former years we have remarked on their admirable arrangement, and the present one is no whit inferior to its predecessors in the interest of its contents and in the conciseness with which every point is expressed.

